# Association Between Vitamin D and Novel SARS-CoV-2 Respiratory Dysfunction – A Scoping Review of Current Evidence and Its Implication for COVID-19 Pandemic

**DOI:** 10.3389/fphys.2020.564387

**Published:** 2020-11-26

**Authors:** Aida Santaolalla, Kerri Beckmann, Joyce Kibaru, Debra Josephs, Mieke Van Hemelrijck, Sheeba Irshad

**Affiliations:** ^1^Translational Oncology and Urology Research, School of Cancer and Pharmaceutical Sciences, King’s College London, London, United Kingdom; ^2^Cancer Research Institute, University of South Australia, Adelaide, SA, Australia; ^3^Department of Medical Oncology, Guy’s and St Thomas’ NHS Foundation Trust, London, United Kingdom; ^4^School of Cancer and Pharmaceutical Sciences, King’s College London, London, United Kingdom

**Keywords:** COVID-19, vitamin D, respiratory dysfunction, SARS-CoV-2, association, scoping review, COVID-19 risk/severity, vitamin D prophylaxis

## Abstract

**Objectives:**

To assess the association between vitamin D deficiency and increased morbidity/mortality with COVID-19 respiratory dysfunction.

**Design:**

Scoping review.

**Data Sources:**

Ovid MEDLINE (1946 to 24 of April 2020) and PubMed (2020 to 17 of September 2020).

**Eligibility Criteria for Selecting Studies:**

A search using the search terms: [(cholecalciferol or ergocalciferol or vitamin D2 or vitamin D3 or vitamin D or 25OHD) and (SARS-CoV-2 or coronavirus or COVID or betacoronavirus or MERS-CoV or SARS-CoV or respiratory infection or acute respiratory distress syndrome or ARDS)]m.p. was conducted on the 24/04/2020 (Search A) and 17/09/2020 (Search B).

**Results:**

91 studies were identified as being concerned with Acute Respiratory Infection (ARI)/Acute Respiratory Distress Syndrome (ARDS) and vitamin D, and 25 publications specifically explored the role of vitamin D deficiency in the development and progression of SARS-CoV-2/COVID-19 related ARDS. Search “A” identified three main themes of indirect evidence supporting such an association. Consistent epidemiological evidence exists linking low vitamin D levels to increased risk and severity of respiratory tract infections. We also report on plausible biological processes supporting such an association; and present weaker evidence supporting the benefit of vitamin D supplementation in reducing the risk and severity of ARIs. Uncertainty remains about what constitutes an appropriate dosing regimen in relation to reducing risk/severity of ARI/ARDS. More recent evidence (Search B) provided new insights into some direct links between vitamin D and COVID-19; with a number of cohort and ecological studies supporting an association with PCR-positivity for SARS-CoV-2 and vitamin D deficiency. The exact efficacy of the vitamin D supplementation for prevention of, or as an adjunct treatment for COVID-19 remains to be determined; but a number of randomized control trials (RCTs) currently underway are actively investigating these potential benefits.

**Conclusion:**

Our rapid review of literature supports the need for observational studies with COVID-19 infected populations to measure and assess vitamin D levels in relation to risk/severity and outcomes; alongside RCTs designed to evaluate the efficacy of supplementation both in preventive and therapeutic contexts. The overlap in the vitamin D associated biological pathways with the dysregulation reported to drive COVID-19 outcomes warrants further investigation.

## Introduction

Vitamin D deficiency affects almost 50% of the population worldwide (Holick, 2007). Its high prevalence is a particularly important public health issue because hypovitaminosis D is an independent risk factor for total mortality in the general population (Melamed et al., 2008). The potential consequences of vitamin D deficiency are wide ranging. It plays a role in the development and progression of chronic diseases such as cardiovascular disease, bone health, autoimmunity, type-2 diabetes, cancer and depression. The less well recognized yet important role of vitamin D includes its effect in modulating the innate and adaptive immune systems, and deficiency of vitamin D is associated with an increased susceptibility to respiratory infections (Dancer et al., 2015; McCartney and Byrne, 2020).

As the pandemic of a novel betacoronavirus known as severe acute respiratory syndrome corona virus 2 (SARS-CoV-2) or 2019 novel coronavirus disease (COVID-19) continues to advance globally, studies on risk factors for intensive care unit admission and mortality are emerging. Early studies from China and Italy report that older age, male sex, smoking status, existing comorbidities or a compromised immune system increase the risk of developing severe and even fatal respiratory disease associated with COVID-19 (Chen et al., 2020). Mortality rates from COVID-19 vary significantly between countries. Countries that lie below 35 degrees North have relatively low mortality rates (e.g., 14.6% of patients in northern latitudes to 0.6% of patients in southern latitudes) (Rhodes et al., 2020). Although this discrepancy has been suggested to be a consequence of the high prevalence of elderly populations in European countries (Bureau, 2020); 35 degrees North is also the latitude above which people do not receive sufficient sunlight to retain adequate vitamin D levels during winter, suggesting a possible role for vitamin D in determining outcomes from COVID-19 (Lips et al., 2019). United Kingdom and United States data from critically ill patients with COVID-19 indicate that people from Black, Asian and minority ethnic (BAME) backgrounds are at a higher risk of developing more severe symptoms than white people (Centre. ICNAaR., 2020). There are also serious concerns regarding the disproportionate impact of COVID-19 on BAME healthcare professionals (Siddique, 2020). Ethnicity is a complex epidemiological entity made up of genetic, social, cultural and behavioral patterns but a growing body of literature indicates that, worldwide, immigrants experience health deterioration after their arrival into their adopted country, and moreover, they have lower vitamin D compared to the native-born population (Holick, 2010; Edwards et al., 2014; Granlund et al., 2016; Martin et al., 2016). Given the prevalence of vitamin D deficiency in BAME populations (Holick, 2007), studies evaluating its role in promoting COVID-19 disease severity are currently underway.

The current scoping review specifically focuses on the potential effects of vitamin D on the risk and severity of COVID-19 associated respiratory dysfunction. We hypothesize that deficiency of vitamin D may be associated with an increased morbidity/mortality of COVID-19 respiratory infection.

## Methods

Following the PRISMA extension for scoping reviews guidelines (Tricco et al., 2018), a initial search was conducted in Ovid MEDLINE, a bibliographic database including references to journal articles from 1946 to date, with the following search terms: [(cholecalciferol or ergocalciferol or vitamin D2 or vitamin D3 or vitamin D or 25OHD) and (SARS-CoV-2 or corona virus or COVID or betacoronavirus or MERS-CoV or SARS-CoV or respiratory infection or acute respiratory distress syndrome or ARDS)].mp. [mp = title, abstract, original title, name of substance word, subject heading word, floating sub-heading word, keyword heading word, organism supplementary concept word, protocol supplementary concept word, rare disease supplementary concept word, unique identifier, synonyms] on the 24 of April 2020 (Search A). Given the rapid turnaround of COVID-19 related literature published following the first peak of the SARS-CoV-2 pandemic, we conduced a second search in PubMed, a bibliographic database that comprises more than 30 million citations for biomedical literature from MEDLINE, life science journals, and online books, with the following search terms: [(cholecalciferol or ergocalciferol or vitamin D2 or vitamin D3 or vitamin D or 25OHD) and (SARS-CoV-2 or corona virus or COVID or betacoronavirus or MERS-CoV or SARS-CoV or respiratory infection or acute respiratory distress syndrome or ARDS)] on the 17 of September 2020 (Search B). There is no published protocol associated with this scoping review.

The inclusion criteria for both searches considered both pediatric and adult publications. Moreover, no restrictions were placed on publication type, with all systematic reviews, narrative reviews, meta-analyses, original research articles (experimental, observational and clinical trials), commentaries, letters, and editorials identified in the MEDLINE search being considered for this review. However, articles concerned with respiratory chronic diseases, such as asthma, and other non-respiratory related pathologies were excluded. Non-English publications, duplicate studies, errata, trial protocols without data and studies not related to human viruses/infections were also excluded. Further exclusions were applied in the second search with only publications with full text available, in human models and published in 2020 included. Studies describing protocols, experimental studies and publications reviewed in the first search were further excluded. Moreover preprints were not included in this review.

Publications were initially screened by title and abstract by three independent reviewers, with potentially relevant studies undergoing a full text review. Following the inclusion and exclusion criteria described above, some studies were further excluded. The included studies were then summarized and classified depending on the topic. Potential bias in some of the observational and interventional studies was identified based on the study design. No formal assessment of quality of selected papers or effect measurement was undertaken given the breadth of publication types being considered and given the scope of the current review.

## Results

**FIGURE 1:**
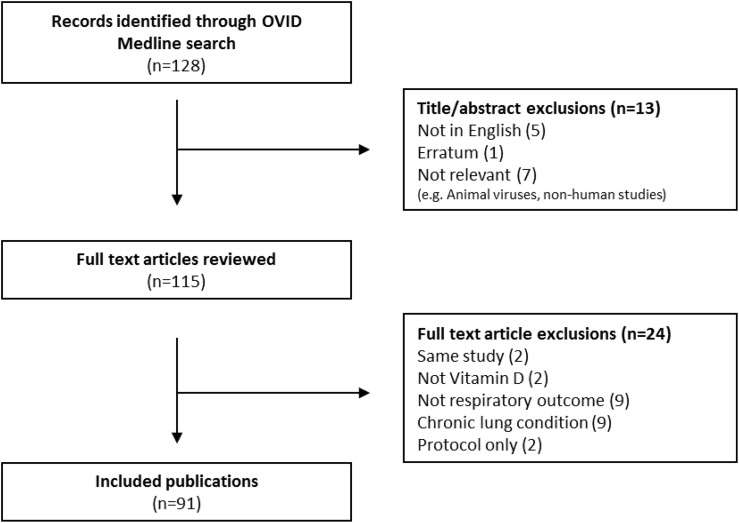
PRISMA diagram presenting the review strategy “A”.

**FIGURE 2:**
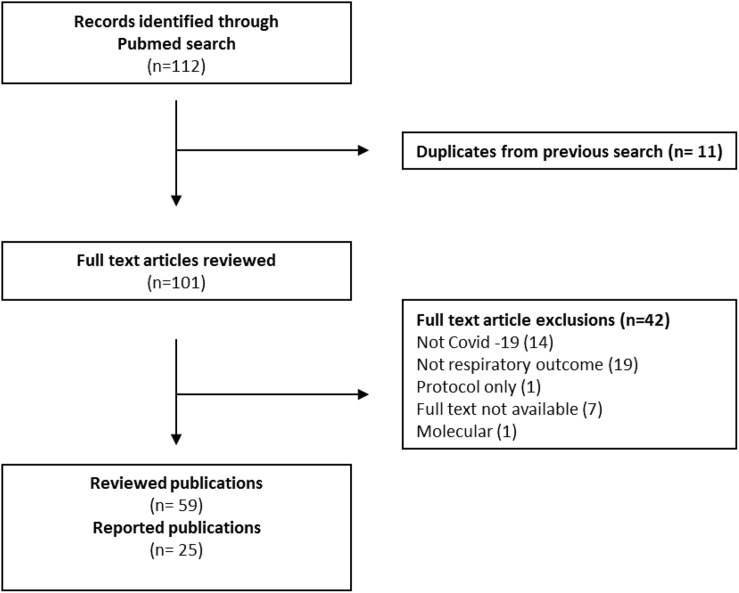
PRISMA diagram presenting the review strategy “B”.

A total of 128 studies were identified from the first search “A.” Full text review was undertaken on 115 articles which were assessed as potentially suitable for inclusion after title and abstract screening. After further exclusion of papers not concerned with COVID-19, ARI/ARDS and vitamin D, the final number of publications included was 91 (Table 1). Figure 1 presents a PRISMA diagram of the review strategy. Reviewed papers on search consisted of 7 systematic reviews with meta-analyses, 13 narrative reviews, 16 randomized trials, 33 observational studies and 5 experimental studies. The remaining (Quesada-Gomez et al., 2020) publications were letters, editorials, commentaries and position statements (Supplementary Table S1). Search “B” identified 112 publications of which 101 were suitable for full inclusion after exclusion of duplicates. After further exclusion for non-Covid related, not relevant and only protocols, 59 publications were reviewed (Supplementary Table S1). Twenty five (8 observational, 4 ecological, 1 interventional non-RCT, 4 Systematic review/meta-analysis/review and 8 editorial) new studies specifically explored the role of vitamin D deficiency in the progression of ARDS in COVID-19 infection (Arnold, 2020; Aygun, 2020; Brenner et al., 2020; Chakhtoura et al., 2020; D’Avolio et al., 2020; Ebadi and Montano-Loza, 2020; Faul et al., 2020; Grant et al., 2020; Haj Bloukh et al., 2020; Hastie et al., 2020; Ilie et al., 2020; Laird et al., 2020; Martineau and Forouhi, 2020; Meltzer et al., 2020; Ohaegbulam et al., 2020; Panagiotou et al., 2020; Quesada-Gomez et al., 2020; Raisi-Estabragh et al., 2020; Silberstein, 2020; Singh et al., 2020; Slominski et al., 2020; Sun et al., 2020; Townsend et al., 2020; Tramontana et al., 2020; Whittemore, 2020) (Tables 2, 3). Figure 2 presents a PRISMA diagram of the review search B.

**TABLE 1:** Overview of the publications from search A included in this scoping review.

**Description of included publications (total = 91)**	**Number**
***Systematic reviews/meta-analyses***	**7**
Target population:	
Children	3
Adults/General	4
Topic:	
Vitamin D supplementation	4
Vitamin D levels/status	3
***Narrative Reviews***	**13**
Target population:	
Children	4
Adults/General	9
Topic:	
COVID-19 specific	3
Acute respiratory distress syndrome	2
Respiratory infections/Lung Disease	8
***Clinical trials/RCTs***	**16**
Target population:	
Neonates/Infants	3
Children	5
Adults/General	8
Topic:	
Respiratory infections	12
Molecular mechanisms	4
***Observational studies***	**33**
Study design	
Cohort	17
Case-Control	11
Cross-sectional	5
Target population:	
Neonates/Infants	9
Children	12
Adults/General	12
Topic:	
Risk of respiratory infections (any/URTI)	8
Risk of ALRI	12
Risk of ARDS	1
Disease severity/Mortality	7
Lung function/Biomarkers	4
***Experimental studies***	**5**
***Other publications***	**17**
Letters	7
Editorials/commentary	8
Position statement/recommendations	2
[COVID-19 specific]	[8]

**TABLE 2:** Overview of the publications from search B included in this scoping review.

**Description of included COVID -19 publications (total = 25)**	**Number**
***Systematic reviews/meta-analyses***	**4**
Topic:	
Vitamin D supplementation	2
Vitamin D levels/status	1
Molecular mechanisms and ARDS	1
***Narrative Reviews/Editorials***	**8**
Topic:	
Bame population and COVID-19	1
Molecular mechanisms and ARDS	3
Vitamin D supplementation and COVID-19 management	4
***Clinical trials/RCTs***	**1**
Topic:	
Vitamin D supplementation in COVID-19 patients	1
***Observational studies***	**12**
Study design	
Cohort	8
Ecological	3
Descriptive cross-sectional	1
Topic:	
Country Incidence and Mortality COVID-19	4
Risk of COVID-19	4
COVID-19 severity/Mortality	4

**TABLE 3:** Ecological, observational and interventional studies addressing Vitamin D levels in COVID-19.

**Authors**	**County**	**Year**	**Study design**	**Population**	**Exposure measure**	**Severity measure**	**Findings: [Vit D deficiency and disease severity]**
Meltzer et al., 2020	United States	2020	Retrospective Cohort	Urban academic medical center Chicago (*n* = 489)	Vit D deficient (1,25-hydroxy-cholecalciferol < 20 ng/ml) measured from 12 m to 14 days before COVID-19 test: combined measure – “likely deficiency” based on most recent Vit D level and Vit D treatment given before test	COVID-19 test positive, based on COVID-19 PCR test	Positive association Patients in the likely Vit D deficient group had higher risk being COVID-19 positive compared with likely sufficient group (RR = 1.77; 1.12–2.81) adjusted for age, sex, race, BMI, comorbidities and employ status.
Brenner et al., 2020	Germany	2020	Retrospective Cohort	Cohort of older adults 50–75 from Saarland, Germany (*n* = 9548) recruited 2000–2002	Vit D insufficiency (30–50 nmol/L) and Vit D deficiency (<30 nmol/L) serum 25(OH)D measured at baseline 2000-02	15 years follow-up (to end of 2016) for respiratory disease mortality. (*n* events = 123)	Positive association Increased risk of resp mortality for Vit D insufficiency (HR 2.1, 1.3-3.2) and Vit D deficiency (HR 3.0, 1.8–5.2) adjusted for age, sex, season, educ, smoking, BMI, PA and fish consumption. Dose response observed. Stronger effect size for resp than all cause, CVD and cancer deaths
Ohaegbulam et al., 2020	United States	2020	Case series (*n* = 4)	Vit D deficient patients with COVID-19 hospitalized at a single hospital in New York (*n* = 4)	Vitamin D supplementation: Cholecalciferol 1000 IU/d or ergocalciferol 50,000 IU/d	Descriptive – all improved and discharge by 14 days; also present biomarker changes (day 0–6)	Positive association Patients that received a high dose of vitamin D supplementation achieved normalization of vitamin D levels and improved clinical recovery evidenced by shorter lengths of stay, lower oxygen requirements, and a reduction in inflammatory marker status
Singh et al., 2020	Study of European countries. Authors from India	2020	Ecological study design	20 selected European countries with data on mean Vit D and COVID-19 cases and deaths	Country level mean serum vit D levels (previously published)	Cases per million and deaths per million population as of April 8 and may 12, 2020	Positive association Using R-squared best fit for linear regression line, authors found stronger (inverse) correlation between mean Vit D and covid death rate at the later time point (post-peak); exponential curve gave better fit; significant correlation for incidence *R* = 0.375 and borderline for deaths *R* = 0.275
Haj Bloukh et al., 2020	Global study, authors from UAE	2020	Descriptive (data on trends in number of cases and fatalities in different countries)	Case numbers and fatality data from selected countries across the world	Mean Vitamin D (just stated as a fact, no measures reported)	Authors just describe patterns/numbers of cases/fatalities from COVID based on data from EDCP to June 3	No association Conclude mean plasma Vit D levels have no influence on fatality by (poor) argument for differences in fatalities for Portugal, Sweden and Switzerland
Whittemore, 2020	United States	2020	Ecological study design	88 countries were selected based on ‘their likelihood of providing reliable data’	Proximity to the equator	Correlation analysis comparing COVID-19 death rates and a country’s latitude. Worldometer website was used to obtain death rates/million for each country	Positive association A significant, positive correlation was found between lower death rates and a country’s proximity to the equator (Pearson *r* = 0.40, *P* < 0.0001, two-tailed *t*-test). Its the first study to document a statistically significant correlation between COVID-19 mortality and a country’s latitude
Sun et al., 2020	China	2020	Retrospective study	*n* = 241, enrolled from February 10 to February 28, 2020	Serum calcium levels	Correlations between serum calcium and clinical outcomes in patients with COVID-19	Positive association Patients with lower serum calcium levels (≤2.0 mmol/L) had worse clinical parameters, higher incidences of organ injury, septic shock, and higher 28-day mortality. serum calcium levels were significantly positively correlated with Vit D levels (*P* = 0.004)
Raisi-Estabragh et al., 2020	United Kingdom	2020	Retrospective Cohort study	4510 UK Biobank aged 40–69 years old participants tested for COVID-19 (positive, *n* = 1326).	Serum 25(OH)-vitamin D levels measured at baseline. Other confounders: age, sex and ethnicity and cardiometabolic factors [diabetes, hypertension, high cholesterol, prior myocardial infarction, smoking and body mass index (BMI)]; 25(OH)-vitamin D; poor diet; Townsend deprivation score; housing (home type, overcrowding) or behavioral factors (sociability, risk taking).	COVID-19 test positive, based on COVID-19 PCR test	No association Male sex, BAME ethnicity, higher BMI, higher Townsend deprivation score and household overcrowding were independently associated with significantly greater odds of COVID-19. Sex and ethnicity differential pattern of COVID-19 was not adequately explained by variations in cardiometabolic factors, 25(OH)-vitamin D levels or socio-economic factors. Serum 25(OH)-vitamin D levels were, on average, higher in White ethnicities than BAME cohorts.
Hastie et al., 2020	United Kingdom	2020	Retrospective Cohort study	348,598 UK Biobank participants. Of these COVID-19 tests was done on 1474 participants and 449 had confirmed COVID-19 infection.	Serum 25(OH)-vitamin D levels measured at baseline Other factors also included sex, month of assessment, Townsend deprivation quintile, household income, self-reported health rating,	COVID-19 test positive, based on COVID-19 PCR test	No association Our findings do not support a potential link between vitamin D concentrations and risk of COVID-19 infection, nor that vitamin D concentration may explain ethnic differences in COVID-19 infection. Vitamin D was associated
					smoking status, BMI quintile, ethnicity, age at assessment, diabetes, systolic blood pressure (SBP), diastolic blood pressure (DBP), and long-standing illness, disability or infirmity.		with COVID-19 infection univariably (OR = 0.99; 95% CI 0.99–0.999; *p* = 0.013), but not after adjustment for confounders (OR = 1.00; 95% CI = 0.998–1.01; *p* = 0.208). Ethnicity was associated with COVID-19 infection univariably (blacks versus whites OR = 5.32, 95% CI = 3.68–7.70, *p*-value < 0.001; South Asians versus whites OR = 2.65, 95% CI = 1.65-4.25, *p*-value < 0.001).
D’Avolio et al., 2020	Switzerland.	2020	Retrospective Cohort study	107 total patients who underwent a nasopharyngeal swab PCR analysis for SARS-CoV-2 and a 25(OH)D measurement, included 27 SARS-CoV-2 PCR-positive. An additional control cohort, 1377 patients with a 25(OH)D measurement during the same period (1 March to 14 April) of 2019 were evaluated.	The vitamin D analysis was required to be conducted within 7 weeks of the SARS-CoV-2 PCR result.	Patients selected for the SARS-CoV-2 PCR analysis had to have symptoms of an acute airway disease (e.g., cough, sore throat, breathing difficulties), with or without fever, feeling of fever, muscle pain, or sudden anosmia or ageusia	Positive association Significantly lower 25(OH)D levels (*p* = 0.004) were found in PCR-positive for SARS-CoV-2 (median value 11.1 ng/mL) patients compared with negative patients (24.6 ng/mL); this was also confirmed by stratifying patients according to age > 70 years.
Ilie et al., 2020	United Kingdom	2020	Ecological study design. Cross-sectional.	20 selected European countries with data on mean of Vitamin D	Mortality caused by COVID- 19 in European countries. Number of cases of COVID-19/1 M population in each of the countries and mortality caused by this disease/1 M population (8th April, 19.00GMT)		Positive association The number of cases/country is affected by the number of tests performed and also by the different measures taken by each country to prevent the spread of infection, and the difference in the number of infected patients in the population will also mean different levels of exposure for the population. Mortality might be a better marker of the number of cases in the population although even that can be influenced by the variations in the approach or management of the disease.
Faul et al., 2020	Ireland	2020	Retrospective Cohort study	33 adult, male, Caucasian patients, over the age of 40 years, who were admitted to Connolly Hospital Blanchardstown for SARS-CoV-2 related pneumonia (four quadrant infiltrates on chest radiograph, with respiratory failure requiring FiO2 greater than 0.4, with SARS-CoV-2 detectable by RT-PCR of nasopharyngeal swab)	Serum 25OHD level on presentation to hospital	Progression to ARDS and required intubation and mechanical ventilation	Positive association In this cohort of thirty three patients, twelve had a baseline 25OHD level less than 30 nmol.l-1. In patients with SARS-CoV-2 related pneumonia a baseline serum 25OHD level less than 30 nmol.l-1 was associated with a hazard ratio (HR) for intubation of 3.19 (95 percent confidence interval, 1.05–9.7), (*p* = 0.03). Twelve patients who progressed to ARDS (mean age 60 years, SD 15) had a lower serum 25OHD level on presentation to hospital (mean = 27, SD = 12 nmol.l −1), compared to the twenty one patients hospitalized with less severe pneumonia who did not progress to ARDS (mean age 56 years, SD 14). Their 25OHD level was 41 nmol.l −1 (SD 19) (*p* = 0.03).
Panagiotou et al., 2020	United Kingdom	2020	Retrospective Cohort study	Serum 25(OH)D levels were measured in 134 (largely Caucasian) inpatients with positive SARS-CoV-2 swab or clinical/radiological diagnosis of COVID-19.	Serum 25OHD level on presentation to hospital	COVID-19 severity and mortality	No association with mortality Positive association with severity. Low serum (25[OH]D) levels in patients hospitalized with COVID-19 are associated with greater disease severity. The majority of COVID-19 inpatients (i.e., 90/134 patients or 66.4%) had vitamin D insufficiency (25–50 nmol/L); 50/134 (37.3%) were deficient (<25 nmol/L), and 29/134 (21.6%) had severe deficiency (≤15 nmol/L). Serum 25(OH)D levels were not associated with mortality [95% CI 0.97 (0.42, 2.23), *P* = 0.94].

Review of search “A” studies found no direct evidence of any association between vitamin D and risk or severity of COVID-19; however, our literature review identified three main themes of indirect evidence supporting an association for vitamin D and SARS-CoV-2 exposure: (1) an association of vitamin D deficiency with increased incidence and severity of respiratory infections (including 12 research studies exploring ARDS/ARI, Table 4), (2) overlapping biological mechanisms of interaction of vitamin D and clinico-pathological features of SARS-CoV-2 infection and (3) the potential role of vitamin D supplementation in prevention of severe acute respiratory tract infections. Moreover, only publications from search B provided new insights into any direct links between vitamin D and COVID-19. Both indirect and direct evidence are discussed in further detail below.

**TABLE 4:** Research studies addressing severity of ARI/ARDS.

**Authors**	**County**	**Year**	**Study design**	**Population**	**Exposure measure**	**Severity measure**	**Findings: [Vit D deficiency and disease severity]**
Pham et al., 2019	Australia	2019	Meta-analysis	Adults (24 studies, 5 for severity)	Vitamin D levels	ARI Severity or mortality	Positive association (OR: 2.46; 95% CI 1.65–3.66)
Dubnov-Raz et al., 2015	Israel	2015	RCT	Healthy adolescents (*n* = 52)	Vitamin D_3_ supplement (2000 IU/d) vs. placebo	Self-reported URTI duration and severity	No association
Martineau et al., 2015	United Kingdom	2015	RCT	Older adults (*n* = 240)	Bimonthly vs. daily vitamin D_3_ supplement (∼10 μg daily)	Self-reported ARI (URI and ALRI) symptom duration	**Inverse** association Higher dose associated with longer duration of URI symptoms
Vo et al., 2018	United States	2018	Cohort study	Infants hospitalized for bronchiolitis (*n* = 1016)	Vitamin D status 25(OH)D < 20 ng/mL	ICU admission and LOS	Positive association (ICU OR 1.72, 95% CI 1.12–2.64) (LOS RR 1.39, 95% CI 1.17–1.65)
Hurwitz et al., 2017	United States	2017	Cohort study	Children hospitalized for viral LRTI (*n* = 90)	Vitamin D status 25(OH)D < 20 nmol/L	ICU admission and ventilation	Positive association (ICU OR 3.29; 95% CI 1.20–9.0) (ventilation OR 11.20 2.23–55.3)
He et al., 2013	United Kingdom	2013	Cohort study	Healthy adults (*n* = 225)	Vitamin D status 25(OH)D < 30 nmol/L	Respiratory illness symptom score	Positive association Vitamin D deficient group had higher URTI symptom scores
Inamo et al., 2011	Japan	2011	Cohort study	Children hospitalized for ALRI (*n* = 28)	Vitamin D status 25(OH)D < 15 nmol/l	Supplemental oxygen and ventilation support	Positive association vitamin D correlated with need for oxygen and ventilator management
Thickett et al., 2015	United States	2015	Cohort study	Adults in ICU (*n* = 1985)	Vitamin D status 25(OH)D < 10 ng/mL	Developed acute respiratory failure (ARDS)	Positive association [OR = 1.84 (95% CI 1.22 to 2.77)]
Barnett et al., 2014	United States	2014	Case-control	Adults in ICU for sepsis or trauma at risk of ARDS (*n* = 240/238)	Vitamin D levels 25(OH)D < 50 v > 75 nmol/L	All-cause mortality	Positive association (High vs. low vitamin D Trauma: HR 0.50, 95% CI 0.35-0.72)
Wayse et al., 2004	India	2004	Case-control	Children (*n* = 150)	Vitamin D status 25(OH)D < 22.5 nmol/L	Hospitalized for severe ALRI	Positive association (High vs. low vitamin D OR: 0.09; 95% CI 0.03–0.24).
McNally et al., 2009.	Canada	2009	Cross-sectional	Hospitalized children (*n* = 197)	Vitamin D levels	ICU admission for ALRI	Positive association (ICU v general ward v controls: 49 v 83 v 87 nmol/L).
Carroll et al., 2011	United States	2011	Cross-sectional	Infants (inpatient, emergency or outpatients) (*n* = 670)	Vitamin D levels	Bronchiolitis score > 12 (chart review)	No association

*Research studies addressing vitamin D and severity of ARI/ARDS.*

## Indirect Evidence for Vitamin D and SARS-CoV-2 Interaction

### Vitamin D Deficiency and Risk and Severity of Acute Respiratory Infections

Patients with severe COVID-19 illness develop dyspnoea and hypoxemia within 1 week after onset of the disease, which may quickly progress to ARDS or end-organ failure, presenting with ARDS in 20% of the COVID-19 patients hospitalized (Zhou et al., 2020). There is reasonable amount of evidence suggesting that higher serum 25-hydroxyvitamin D [25(OH)D] concentrations reduce the risk of respiratory tract infections across all age groups (Wayse et al., 2004; McNally et al., 2009; Carroll et al., 2011; Inamo et al., 2011; Laaksi, 2012; He et al., 2013; Jolliffe et al., 2013; Barnett et al., 2014; Dancer et al., 2015; Monlezun et al., 2015; Thickett et al., 2015; Confalonieri et al., 2017; Hurwitz et al., 2017; Umehara et al., 2017; Vo et al., 2018; Iyer and Bansal, 2019; Pham et al., 2019; Zhang et al., 2019; McCartney and Byrne, 2020). A number of these studies specifically implicate vitamin D deficiency as an independent risk factor for ARI/ARDS (Jolliffe et al., 2013; Barnett et al., 2014; Monlezun et al., 2015; Confalonieri et al., 2017; Umehara et al., 2017; Iyer and Bansal, 2019) (Table 4). McCartney reported on a meta-analysis of 20,966 subjects that individuals with vitamin D levels < 20 ng/ml had an increased risk of community-acquired pneumonia [OR (95% CI) = 1.64 (1.00, 2.67)] (McCartney and Byrne, 2020). Similarly, in new-born an association between subclinical vitamin D and increased risk of acute lower respiratory infections (ALRI) has also been observed (Karatekin et al., 2009; Carroll et al., 2011). Among adults a meta-analysis of 19 observational studies reported that serum 25(OH)D concentration is inversely associated with risk and severity of ARI [pooled OR (95% CI) were 1.83 (1.42–2.37) and 2.46 (1.65–3.66), respectively] (Pham et al., 2019). An inverse association was observed in a US cohort (*n* = 1985) of critically ill adults admitted to ICU between vitamin D levels and acute respiratory failure [adj. OR < 10 vs. 30+ ng/mL (95% CI) = 1.84 (1.22–2.77)]. Similarly, an inpatients cohort showed vitamin D levels were strongly associated with 90 days mortality from fatal acute respiratory failure [adj. OR < 20 vs. 30+ ng/mL (95% CI) = 2.48(1.45–4.25)] (Thickett et al., 2015), a finding supported by Umehara et al. (2017). Brenner et al. (2020) observed an association between Vitamin D deficiency and increased mortality from respiratory diseases in a cohort of older adults with 15 years of follow up. Inamo et al. (2011) observed that significantly more children with ALRI requiring supplementary oxygen and ventilator management were vitamin D deficient. In summary, there is robust evidence for the immunomodulatory properties of vitamin D in influencing ALRI disease severity (McNally et al., 2009; Chaabouni et al., 2020); hereby providing proof-of-principle for its involvement in progression of COVID-19 pathogenesis.

### Biological Mechanisms of Interaction of Vitamin D and Clinico-Pathological Features of SARS-CoV-2 Infection

The pathogenic mechanisms of COVID-19 disease have not been completely elucidated yet. However, a number of dysregulated pathways are being increasingly recognized as directly related to COVID-19 morbidity and mortality (Jakovac, 2020; Misra et al., 2020; Panarese and Shahini, 2020). These include the role of renin-angiotensin pathways in viral entry into alveolar cells and subsequent acute lung injury, dysregulated immune responses and activation of coagulation pathways (Quesada-Gomez et al., 2020). Interestingly, our literature review identified a number of studies/reviews (*n* = 24) providing some insight into the mechanistic explanations for the observed epidemiological relationship between vitamin D deficiency and increased risk/severity of respiratory infections (Ginde et al., 2009; Laaksi, 2012; Pfeffer and Hawrylowicz, 2012; He et al., 2013; Parekh et al., 2013; Lambert et al., 2014; Dancer et al., 2015; Greiller and Martineau, 2015; Vargas Buonfiglio et al., 2017; Xu et al., 2017; Jolliffe et al., 2018; Miraglia Del Giudice et al., 2018; Iyer and Bansal, 2019; Aygun, 2020; Brenner et al., 2020; Carter et al., 2020; Jakovac, 2020; Kakodkar et al., 2020; Maes et al., 2020; McCartney and Byrne, 2020; Misra et al., 2020; Molloy and Murphy, 2020; Panarese and Shahini, 2020; Quesada-Gomez et al., 2020; Tian and Rong, 2020; Zheng et al., 2020).

Studies and narrative reviews suggest that entry of SARS-CoV-2 infection into alveolar epithelial cells, triggered by binding of the virus surface spike (S)-protein to the angiotensin converting enzyme 2 (ACE2) receptor, may lead to dysregulation of the renin-angiotensin system (RAS) resulting in acute lung injury due to toxic over-accumulation of angiotensin II (Ang II) in alveolar cells (Pfeffer and Hawrylowicz, 2012; Arnold, 2020; Carter et al., 2020; Jakovac, 2020; Kakodkar et al., 2020; Misra et al., 2020; Panarese and Shahini, 2020; Tian and Rong, 2020). Evidence suggests that vitamin D deficiency causes over-activation of the pulmonary RAS, providing a possible explanation for the observed inverse relationship between blood pressure and serum 25(OH)D levels (Parekh et al., 2013; Xu et al., 2017). Vitamin D may exert protective effects on acute lung injury, at least in part, by regulating the balance between the expression of members of the RAS (Xu et al., 2017). It is therefore plausible that vitamin D deficiency may exacerbate dysregulation of the pulmonary RAS induced by SARS-CoV-2 infection.

ACE2 receptor required for virus entry, is expressed by endothelial cells and its density in each tissue may correlate with the severity of the disease in that tissue (Jia et al., 2005; Gheblawi et al., 2020; Perico et al., 2020; Xu et al., 2020). Endothelial dysfunction is a common feature of the clinical manifestations observed in COVID-19 (e.g., high blood pressure, thrombosis, kidney disease, pulmonary embolism, Kawasaki vasculitic disease, cerebrovascular and neurological disorders) (Sardu et al., 2020b). A systematic and comprehensive evaluation of literature by Sardu et al. (2020b) report that in the hospitalized patients with COVID-19 disease the worsening disease severity, including ICU admissions, could be played by endothelial dysfunction that is more enhanced in patients with co-morbidities. Study by. Guan et al. reported that 23.7% 23.7% of hypertensive patients had disease severity (vs. 13.4% of normotensive subjects), and that 35.8% (vs. 13.7%) reached the composite endpoint of ICU admission, mechanical ventilation and death (Guan et al., 2020). Paradoxically, many of the therapies for pre-existing cardiovascular diseases can also increase the ACE2 levels resulting in a negative outcome regard to infectivity and outcome of COVID−19. However, given the high incidence of cardiac injury and adverse outcomes in hypertensive patients during the COVID-19 pandemic, experts do not recommend that hypertensive patients should discontinue and/or switch therapies during COVID-19 infection. Interestingly, vitamin D treatment has been shown to inhibit ACE2 expression in the kidneys (Lin et al., 2016; Ali et al., 2018), and thereby could prevent COVID-19 entry into the cell in patients with pre-existing co-morbidities. Additionally, Zhou et al. recently showed a significant association between levels of D-dimer > 1 μg/mL and COVID-related death [OR (95 %CI):18.42 (2.64–128.55)], with coagulation dysfunction associated with fatal ARDS in COVID-19 patients (Sardu et al., 2020c; Zhou et al., 2020). Vitamin D and its associated metabolites are implicated in the regulation of thrombosis-related pathways by modulation of genes such as those related to angiogenesis (Chen et al., 2020). It has been proposed that this is likely to be the mechanism of action for the increased risk of thrombotic events associated with a vitamin D deficient state (Tian and Rong, 2020). These studies strongly support investigation of vitamin D replacement in patients with pre-existing co-morbidities.

Genetic studies have found a polymorphism in the vitamin D receptor (VDR) gene associated with upper respiratory tract infections in children and adults (Jolliffe et al., 2018). Dysregulated immune responses associated with excessive and prolonged cytokine/chemokine responses known as a ‘cytokine storm’, seem to be directly related to worse outcomes in COVID-19 (Carter et al., 2020; Slominski et al., 2020; Tian and Rong, 2020). All cells of the immune system have been shown to express the VDR, including T cells, and literature suggests that it is now well accepted that 1,25(OH)2D and vitamin D are important immune system regulators (Laaksi, 2012; Parekh et al., 2013; Greiller and Martineau, 2015; Miraglia Del Giudice et al., 2018; Iyer and Bansal, 2019; Maes et al., 2020; Misra et al., 2020; Tian and Rong, 2020). Vitamin D has been linked to innate immunity through identification of the cationic antimicrobial peptide cathelicidin as a vitamin D target gene and VDR upregulation in monocytes. Previous studies have proposed cathelicidin activation as a potential mechanism driving vitamin D protective effects on respiratory tract infections (Laaksi, 2012; Lambert et al., 2014; He et al., 2016; Vargas Buonfiglio et al., 2017; Molloy and Murphy, 2020), e.g., in tuberculosis (Ginde et al., 2009; Molloy and Murphy, 2020). A cross-sectional analysis in a mostly African-American population showed that lung function decrements associated with low cathelicidin were greatest in individuals with low vitamin D levels (Lambert et al., 2014). Additionally, vitamin D is involved in downregulating the production of pro-inflammatory cytokines [tumor necrosis factor α (TNF α), interleukin-6 (IL-6), interleukin-8 (IL-8), interleukin-12 (IL-12), and interferon-gamma (IFN-γ)] which contribute to the cytokine storm (Pfeffer and Hawrylowicz, 2012; He et al., 2013; Barnett et al., 2014; Zhang et al., 2019; Aygun, 2020; Maes et al., 2020; Orrù et al., 2020; Tian and Rong, 2020). Downregulation of IFNγ and IL-6 inflammatory responses have been reported as being negative prognostic indicators in critically ill ventilated patients including those with COVID-19 (McCartney and Byrne, 2020). Other vitamin D functions such as the musculoskeletal properties can be also potentially beneficial in the context of COVID-19 (Tramontana et al., 2020). While the data are far from conclusive in attributing a role for vitamin D in influencing the risk and severity of COVID-19, collectively these studies provide a strong rationale for more pre-clinical and clinical research for further exploration.

### Vitamin D Supplementation in Relation to Acute Respiratory Tract Infections

Over the years numerous randomized controlled trials (RCTs) have evaluated whether vitamin D supplementation can decrease the risk of acute respiratory tract infections (ARTI) and its severity. Such studies have yielded conflicting results. Pediatric RCTs exploring the association of vitamin D supplementation with reduction of ARIs have mainly reported no association (Ginde et al., 2009; Camargo et al., 2012; Maxwell et al., 2012; Goldring et al., 2013; Grant et al., 2015, 2016; Xiao et al., 2015; Omand et al., 2017; Somnath et al., 2017; Das et al., 2018; Lee et al., 2018; Hauger et al., 2019; Loeb et al., 2019). A large cohort study assessing the effects on serum vitamin D levels following oral vitamin D supplementation in children (0–5 years) found no association between either vitamin D levels or supplementation on health-service utilization (HSU) for upper respiratory tract infections (URTIs) (Omand et al., 2017). Similarly, a meta-analysis of 7 RCTs presented a non-significant association between vitamin D supplementation and reduced risk of ARIs in children [RR (95%CI): 0.79 (0.55–1.13)] (Xiao et al., 2015). Pre-natal and infant studies have also found no effect (Goldring et al., 2013; Grant et al., 2016). The relevance of these studies to adults, however, is unclear.

Several studies undertaken in adults show inconsistent results especially in the elderly population (Murdoch et al., 2012; He et al., 2013; Jolliffe et al., 2013; Dubnov-Raz et al., 2015; Martineau et al., 2015, 2017, 2019; Vargas Buonfiglio et al., 2017; Aloia et al., 2019; Camargo et al., 2019; Kakodkar et al., 2020). Whilst a systematic review of 39 studies including observational studies and RCTs reported inconsistent results for vitamin D supplementation for prevention of ARI (Jolliffe et al., 2013). Moreover, a meta-analysis in 2017 of individual data from 10,933 study subjects in 25 RCTs, showed an 12% overall protective effect of oral vitamin D3 supplementation against bacterial and viral ARTI [OR(95%CI): 0.88 (0.81–0.96)]. Benefit was greater in those receiving daily or weekly vitamin D (*P* for interaction = 0.05) and in those with serum 25(OH)D levels < 25 nmol/L [adjusted OR (95%CI): 0.30(0.17 – 0.53)] (Martineau et al., 2017, 2019). A UK study examined the effect of 14 weeks of vitamin D3 supplementation (5000 IU/d) during winter in male athletes, on antimicrobial proteins in saliva. The authors observed increases over time in saliva secretory immunoglobulin A (SIgA) and the antimicrobial peptide cathelicidin in the vitamin D3 group (both *P* = 0.03), suggesting that these changes may increase resistance to respiratory infections (He et al., 2013).

A number of RCTs studies in mechanically ventilated, critically ill patients with high dose supplementation (250,000–500,000 IU vitamin D) have proven to be safe and observed association with decreased of hospital length stay and increase of hemoglobin serum levels (Perron and Lee, 2013; Han et al., 2016; Putzu et al., 2017; Smith et al., 2018; Ebadi and Montano-Loza, 2020). Additionally, it is important to mention the diversity observed among the RCTs with respect to supplementation regimen (monthly/weekly/daily), the intake dose (1000–5000 IU/d, 14000/w, 12000–200000 IU/m, or 300000 IU single dose) and the duration of the intervention (1 year - > 3 years) make it difficult to have clear cross-study comparisons. Moreover, baseline vitamin D levels were not assessed in many studies, and many cases vitamin D intake and respiratory events were self-reported. However, based on these studies although vitamin D supplementation can be concluded to be preventive for ARIs in adults, uncertainty around therapeutic benefit/dosing required remains.

## Direct Evidence for Vitamin D and SARS-CoV-2 Interaction

A more up-to-date search (Search B) identified a number of studies providing a more direct link or evidence for an association of vitamin D and COVID-19. These are summarized below.

### Vitamin D Deficiency and COVID-19 Incidence/Disease Severity

Human requirement for vitamin D, assessed by measuring 25(OH)D levels in blood plasma or serum, is achieved primarily through the synthesis of this pre-hormone in the skin during exposure to ultraviolet B (UVB) radiation, with minor contribution from diet, year round. This process is negatively influenced by skin pigmentation. A level of 20–50 nanograms/milliliter is considered adequate for healthy people. A level less than 12 ng/mL indicates vitamin D deficiency (Holick, 2007). Vitamin D deficiency have reported to be associated with PCR-positivity for SARS-CoV-2 individuals in two different cohorts. D’Avolio et al. observed significantly lower 25(OH)D levels (*p* = 0.004) in PCR-positive patients for SARS-CoV-2 (median value 11.1 ng/mL) compared with negative patients (24.6 ng/mL) in a cohort Study in Switzerland suggesting a potential role of the vitamin D in the COVID-19 infection (D’Avolio et al., 2020). Similar results were observed in a healthy workers cohort in Chicago where the vitamin D deficient group had a higher risk for COVID-19 positivity compared with the control group (RR = 1.77; 1.12–2.81) (Meltzer et al., 2020). This positive association was also observed in a large US cohort study (*n* = 191,779) where SARS-CoV-2 positivity was strongly and inversely associated with circulating 25(OH)D levels after adjusting for latitudes, races/ethnicities, both sexes, and age ranges (Kaufman et al., 2020). Moreover, Faul et al. (2020) presented a small cohort of patients with PCR-positive SARS-CoV-2 related pneumonia, where a baseline serum 25OHD level less than 30 nmol.l-1 was associated with a hazard ratio (HR) for intubation of 3.19 (95 percent confidence interval, 1.05–9.7), (*p* = 0.03) suggesting that low 25OHD level, contributes to severe disease and progression to ARDS in some patients infected with SARS-CoV-2 (Faul et al., 2020). In line with this results, low serum 25[OH]D levels in patients hospitalized with COVID-19 was associated with greater disease severity but not with mortality in a United Kingdom COVID-19 patients cohort (Panagiotou et al., 2020). Serum calcium levels are well recognized to be significantly positively correlated with vitamin D levels; and higher incidences of organ injury, septic shock, and higher 28-day mortality have been observed in COVID-19 patients with lower calcium levels (Sun et al., 2020). Collectively these results provide a strong rationale for further exploration of the potential role of vitamin D and its supplementation where deficient in COVID-19 infection; as well as its role as a predictive biomarker for COVID-19 severity (Weir et al., 2020).

Study by Whittemore (2020) reported a statistically significant correlation between lower COVID-19 death rates and a country’s latitude, suggesting a correlation between sunlight exposure and reduced mortality. In support of this, two other ecological studies have explored the correlation between mean vitamin D levels and incidence and COVID-19 related mortality across selected European countries (Ilie et al., 2020; Singh et al., 2020). Both studies found a strong inverse correlation between mean vitamin D level in various European countries and COVID-19 mortality (Ilie et al., 2020; Singh et al., 2020) or COVID-19 incidence (Ilie et al., 2020) which was also supported by the results of the systematic review by Laird et al. (2020). However, ecological approaches are at risk of potential confounding effects given the variation in measurements of a dependent variable such as number of detected COVID-19 cases; which for example may be significantly different from the number of true cases primarily due to differences in local screening policies (Garg et al., 2020; Gostic et al., 2020; Maruotti et al., 2020). Additionally, none of the above-mentioned studies report the duration of time between the identification of first cases in a country and the reported death rates are unknown. It is possible that death rates in a country where cases began to appear recently will appear lower than those in countries fighting the virus for a longer time. For example, Portugal (39 nmol/L) and Sweden (73.5 nmol/L) were reported as having the lowest or highest mean serum vitamin D levels for the populations respectively. However, the number of fatalities as of 16/10/2020 in Sweden are higher than Portugal, contradicting the conclusions drawn by the authors. Interestingly, two cohort studies exploring the potential association of vitamin D deficiency and the high incidence of COVID-19 in BAME individuals have not reported positive correlations between the two (Hastie et al., 2020; Raisi-Estabragh et al., 2020). Raisi-Estabragh et al. (2020) specifically explore whether cardiometabolic exposures, including vitamin D, socio-economic, lifestyle and behavioral exposures could explain the differential patterns of COVID-19 incidence observed in male and BAME patient populations. The authors could not conclude that sex and ethnicity differential pattern of COVID-19 were explained adequately by variations in cardiometabolic factors, 25(OH)-vitamin D levels or socio-economic factors, however, a sensitivity analysis specifically exploring the interaction of BAME ethnicity and vitamin D levels was not conducted. Hastie et al. (2020) found no association between plasma 25(OH)D concentrations and COVID-19 infection in the UK biobank (Faul et al., 2020) nor in the BAME population after adjusting for potential confounders. However, the results need to be interpreted with caution due to the over adjustment of the analyses including mediators of disease and the potential error in the outcome assessment and vitamin D levels (Grant and McDonnell, 2020; Roy et al., 2020). Both studies found significantly lower levels of vitamin D in the COVID-19 positive population and both studies assessed baseline vitamin D levels (samples taken between 2006 and 2010). Some authors have suggested that the interaction between BAME ethnicity, worse COVID-19 outcomes and vitamin D deficiency is mediated by other factors such as obesity and socioeconomic factors (Chakhtoura et al., 2020; Grant et al., 2020; Muscogiuri et al., 2020; Orrù et al., 2020; Sardu et al., 2020a; Townsend et al., 2020). These hypotheses need to be further investigated in observational studies.

We can conclude here that there is a potential benefit of vitamin D supplementation for prevention and treatment of COVID-19 patients, however the true association between mean plasma 25(OH)D levels and COVID-19 outcomes remains unclear and needs to be further explored with well-designed RCTs.

### Vitamin D Supplementation for COVID-19 Prevention/Treatment

Considering that adverse events related to vitamin D toxicity are rare (Kimball et al., 2017; McCartney and Byrne, 2020; Slominski et al., 2020), experts have since recommended vitamin D supplementation of 10,000 IU/d of vitamin D3 for a few weeks to rapidly raise 25(OH)D levels, followed by 5000 IU/d, with the goal of raising 25(OH)D levels to >40–60 ng/mL (100–150 nmol/L) for populations at risk of influenza and/or COVID-19 (Aloia and Li-Ng, 2007; Martineau et al., 2009, 2019; Urashima et al., 2010; Alexander et al., 2020; Chakhtoura et al., 2020; Siuka et al., 2020; Tramontana et al., 2020; van der Meulen, 2020). For treatment of COVID-19 patients, higher vitamin D3 doses were recommended by the authors (Ebadi and Montano-Loza, 2020; Grant et al., 2020; Liu et al., 2020; Slominski et al., 2020). A recent case series publication on 4 COVID-19 patients with vitamin D deficiency reported that all 4 patients treated with cholecalciferol 1000 IU/d or ergocalciferol 50,000 IU/d had a good outcome and were discharged within 14 days; with biomarker changes from day 0–6 observed (Ohaegbulam et al., 2020). Caccialanza et al. (2020) have also recently published a protocol implementation for early nutritional supplementation of non-critically ill patients hospitalized with COVID-19 in Italy, whereby cholecalciferol is promptly prescribed according to blood tests results [50,000 IU/wk. if 25(OH)D < 20 ng/mL; 25,000 IU/wk. if 25(OH)D 20 to <30 ng/mL]. Beigmohammadi et al. (2020) RCT for patients with COVID-19 admitted to intensive care will supplement the intervention arm with a number of vitamins including a single dose of Vitamin D 600,000 IU to assess severity and mortality rate. Quesada-Gomez et al. (2020) suggested that the stimulation of VDR in patients with SARS-CoV-2 infection, may reduce ARDS due to VDR’s capacity as a negative regulator of renin-angiotensin system in alveolar cells and recommended oral calciferol supplementation as a therapy approach. However, according to our literature review the exact efficacy of the above-mentioned measures for prevention of, or as an adjuvant treatment for COVID-19 remains to be determined.

The results of 9 ongoing RCTs (26 registered) on vitamin D are early awaited as they will help to elucidate the role of vitamin D in COVID-19 infection and its potential benefit in prevention and treatment of COVID-19 disease (Dos Santos, 2020). These include: 4 RCTs exploring survival rate on COVID-19 patients [NCT04334005 (single dose of 25,000 UI of vitamin D), ZnD3-CoVici (NCT0435149) (2000 IU per day for 2 months), CoVitTrial (NCT04344041) (single dose of cholecalciferol 400,000 IU compared to a single dose of 50,000 IU), COVIDIOL (NCT04366908)] [Calcifediol or not (calcifediol in soft capsules: 0.532 mg on the day of admission and 0.266 mg on day 3 and 7 and then weekly until discharge or ICU admission)]; 2 studying association with COVID symptoms and hospitalization [LEAD COVID-19 (50,000 IU of vitamin D, weekly for 2 weeks), HELPCOVID-19 (NCT04335084) (No dose available)] and 1 study exploring the potential preventive effect of calcifediol in healthy population [(NCT04386850) (25 mcg of 25OHD3 once daily at bedtime for 2 months and the control group will receive placebo daily for 2 months)] (Quesada-Gomez et al., 2020). Moreover, Martineau (2020) have launched a RCT (COVIDENCE UK) which aim to recruit 12000 individuals to investigate how diet and lifestyle factors might influence transmission of SARS-CoV-2, severity of COVID-19 symptoms, speed of recovery, and any long-term effects.

## Conclusion

The spread of novel SARS-CoV-2 virus, resulting in COVID-19 has attracted renewed interest in vitamin D; being dubbed the ‘magic-bullet’ or ‘cure’ for COVID-19. The aim of this publication was to assess the current evidence between vitamin D deficiency and increased morbidity/mortality with COVID-19 respiratory dysfunction. Lack of standardization of the clinical cut-offs for diagnosing hypovitaminosis D, the heterogeneity in supplementation regimes and duration, and the many self-reported measures of exposures and outcomes in RCTs make robust metanalyses difficult. Collectively, our scoping review highlights that the epidemiological studies exploring an association between circulating levels of 25[OH]D (the biomarker of vitamin D status) and incidence and severity of COVID-19 show a positive association but are mainly observational studies and limited in number. However, two ecological studies have observed inverse correlations between vitamin D status and incidence and mortality of COVID-19 (Ilie et al., 2020; Singh et al., 2020), also supported by other studies including a systematic review (D’Avolio et al., 2020; Kaufman et al., 2020; Laird et al., 2020; Meltzer et al., 2020; Panagiotou et al., 2020). Supplementation in a small case study of COVID-19 patients had a positive outcome (Grant and McDonnell, 2020), supporting previous observations in ICU mechanically ventilated patients (Perron and Lee, 2013; Han et al., 2016; Smith et al., 2018). Based on the above described studies vitamin D supplementation can be concluded to be preventive for ARIs in adults, but uncertainty around therapeutic benefit/dosing required remains.

In conclusion, the evidence presented here supports further observational and multicenter well-designed randomized clinical studies on the role of vitamin D in preventing and/or treating COVID-19 infections. The overlap in the vitamin D associated biological pathways with the dysregulation reported to drive COVID-19 outcomes warrants further investigation, as does the role that vitamin D levels at time of presentation of COVID-19 may have as predictive biomarkers of disease severity. It is important for future work that implications for at-risk populations (BAME, elderly, pregnant, immunocompromised, obese) are taken into consideration when defining the vitamin D dose regimens and duration. Moreover, clinical trials focused on inpatients presenting with COVID-19 are needed to provide a better understanding of the potential of vitamin D as an adjunct treatment.

## Author’s Note

AS and SI affirm that the manuscript is an honest, accurate, and transparent account of the study being reported; that no important aspects of the study have been omitted; and that any discrepancies from the study as planned (and, if relevant, registered) have been explained.

## Author Contributions

AS and SI conceived the study idea. AS, JK, and KB performed the literature search. AS, KB, and JK acquired the data and performed the credibility assessments. AS, KB, and SI developed the core criteria used in this study. AS wrote the first draft of the manuscript. AS, KB, DJ, MV, and SI critically revised the manuscript. AS and SI had full access to all of the data in the study, and took responsibility for the integrity of the data and the accuracy of the data analysis. AS and SI were the guarantors. AS attested that all listed authors meet authorship criteria and that no others meeting the criteria have been omitted. All the authors contributed to the article and approved the submitted version.

## Conflict of Interest

The authors declare that the research was conducted in the absence of any commercial or financial relationships that could be construed as a potential conflict of interest.

## Supplementary Material

Supplementary Table 1 Summary of all the articles included in this review. Table subdivided in 5 subtables one per type of publication included in the scoping review.

## Abbreviations

25(OH)D: 25-hydroxy vitamin D
ALRI: acute lower respiratory infection
ARI: acute respiratory infection
ARDS: acute respiratory distress syndrome
ALI: acute lung injury
ACEI ACEII: angiotensin-converting enzymes I and II
BAME, black: asian and minority ethnic backgrounds
RCTs: Randomized Clinical Trials
RAS: renin-angiotensin system
RTI: respiratory tract infection
SARS-CoV-2: severe acute respiratory syndrome coronavirus 2.
