# Supplementary material for: Association Between Vitamin D and Novel SARS-CoV-2 Respiratory Dysfunction – A Scoping Review of Current Evidence and Its Implication for COVID-19 Pandemic
Source: Front Physiol. 2020 Nov 26;11:564387. doi: 10.3389/fphys.2020.564387 (PMC7726316; doi:10.3389/fphys.2020.564387)
Supplement: Supplementary Table 1 — Summary of all the articles included in this review. Table subdivided in 5 subtables one per type of publication included in the scoping review. [file Table_1.docx]

## Appendix

Supplementary Table 1. Summary of all the articles included in this review. Table subdivided in 5 subtables one per type of publication included in the scoping review.

| **Type** | **Title** | **Authors** | **Year** |
| --- | --- | --- | --- |
| Systematic review/meta-analysis | Vitamin D supplementation to prevent acute respiratory infections: individual participant data meta-analysis. | Martineau (103)AR, Jolliffe DA, Greenberg L,  et al | 2019 |
| Systematic review/meta-analysis | Acute Respiratory Tract Infection and 25-Hydroxyvitamin D Concentration: A Systematic Review and Meta-Analysis. | Pham H, Rahman A, Majidi A, Waterhouse M, Neale RE(55) | 2019 |
| Systematic review/meta-analysis | Vitamin D as an adjunct to antibiotics for the treatment of acute childhood pneumonia. | Das RR, Singh M, Naik SS(95) | 2018 |
| Systematic review/meta-analysis | Vitamin D supplementation to prevent acute respiratory tract infections: systematic review and meta-analysis of individual participant data. | Martineau, Jolliffe, L Hooper, et al(102) | 2017 |
| Systematic review/meta-analysis | Vitamin D supplementation for the prevention of childhood acute respiratory infections: a systematic review of randomised controlled trials. | Xiao L, Xing C, Yang Z, et al.(91) | 2015 |
| Systematic review/meta-analysis | Vitamin D in the prevention of acute respiratory infection: systematic review of clinical studies. | Jolliffe DA, Griffiths CJ Martineau AR(44) | 2013 |
| Narrative Review | A Comprehensive Literature Review on the Clinical Presentation, and Management of the Pandemic Coronavirus Disease 2019 (COVID-19). | Kakodkar P, Kaka N, Baig MN(69) | 2020 |
| Narrative Review | Rheumatologists' perspective on coronavirus disease 19 (COVID-19) and potential therapeutic targets. | Misra DP, Agarwal V, Gasparyan AY, Zimba O(61) | 2020 |
| Narrative Review | Targeting Vitamin D Deficiency to Limit Exacerbations in Respiratory Diseases: Utopia or Strategy With Potential? | Maes K, Serre J, Mathyssen C, Janssens W, Gayan-Ramirez G(72) | 2020 |
| Narrative Review | Evidence that Vitamin D Supplementation Could Reduce Risk of Influenza and COVID-19 Infections and Deaths. | Grant WB, Lahore H, McDonnell SL, et al(33) | 2020 |
| Narrative Review | What do we know about optimal nutritional strategies in children with paediatric acute respiratory distress syndrome? | Iyer R, Bansel A(47) | 2019 |
| Narrative Review | Acute respiratory distress syndrome. | Confalonieri M,Salton F, Fabiano F(46) | 2017 |
| Narrative Review | Modulation of the immune response to respiratory viruses by vitamin D. | Greiller C , Martinaeu A(73) | 2015 |
| Narrative Review | Vitamin D deficiency and acute lower respiratory infections in children younger than 5 years: identification and treatment. | Larkin A, Lassetter J(134) | 2014 |
| Narrative Review | Better new-born vitamin D status lowers RSV-associated bronchiolitis in infants. | Maxwell C, Carbone E & Wood R(96) | 2012 |
| Narrative Review | Vitamin D and lung disease. | Pfeffer P & Hawryloxicz C(68) | 2012 |
| Narrative Review | Vitamin D and respiratory infection in adults. | Laaksi I(42) | 2012 |
| Narrative Review | Child nutrition and lower respiratory tract disease burden in New Zealand: a global context for a national perspective. | Grant C, Wall C, Gibbons M, et al (135) | 2011 |
| Narrative Review | Vitamin D, respiratory infections, and asthma. | Ginde AA, Mansbach J, Camargo C(78) | 2009 |
| Systematic review/meta-analysis/review | Current State of Evidence: Influence of Nutritional and Nutrigenetic Factors on Immunity in the COVID-19 Pandemic Framework | Galmés S, Serra F, Palou A. | 2020 |
| Systematic review/meta-analysis/review | A Single Large Dose of Vitamin D Could be Used as a Means of Coronavirus Disease 2019 Prevention and Treatment | Liu G, Hong T, Yang J. | 2020 |
| Systematic review/meta-analysis/review | The importance of vitamin d metabolism as a potential prophylactic, immunoregulatory and neuroprotective treatment for COVID-19 | Xu Y, Baylink DJ, Chen CS, Reeves ME, Xiao J, Lacy C, Lau E, Cao H. | 2020 |
| Systematic review/meta-analysis/review | Could Vitamins Help in the Fight Against COVID-19? | Jovic TH, Ali SR, Ibrahim N, Jessop ZM, Tarassoli SP, Dobbs TD, Holford P, Thornton CA, Whitaker IS. | 2020 |
| Systematic review/meta-analysis/review | Early Nutritional Interventions with Zinc, Selenium and Vitamin D for Raising Anti-Viral Resistance Against Progressive COVID-19 | Alexander J, Tinkov A, Strand TA, Alehagen U, Skalny A, Aaseth J. | 2020 |
| Systematic review/meta-analysis/review | Natural history of COVID-19 and current knowledge on treatment therapeutic options | Dos Santos WG. | 2020 |
| Systematic review/meta-analysis/review | Inhibitory effects of Vitamin D on inflammation and IL-6 release. A further support for COVID-19 management? | Orrù B, Szekeres-Bartho J, Bizzarri M, Spiga AM, Unfer V. | 2020 |
| Systematic review/meta-analysis/review | Immunologic Effects of Vitamin D on Human Health and Disease | Charoenngam N, Holick MF. | 2020 |
| Systematic review/meta-analysis/review | Vitamin D and Inflammation: Potential Implications for Severity of Covid-19 | Laird E, Rhodes J, Kenny RA. | 2020 |
| Systematic review/meta-analysis/review | Vitamin D receptor stimulation to reduce acute respiratory distress syndrome (ARDS) in patients with coronavirus SARS-CoV-2 infections: Revised Ms SBMB 2020_166 | Quesada-Gomez JM, Entrenas-Castillo M, Bouillon R. | 2020 |
| Systematic review/meta-analysis/review | Does Vitamin D play a role in the management of Covid-19 in Brazil? | Ribeiro H, Santana KVS, Oliver SL, Rondó PHC, Mendes MM, Charlton K, Lanham-New S. | 2020 |
| Systematic review/meta-analysis/review | Strengthening the Immune System and Reducing Inflammation and Oxidative Stress through Diet and Nutrition: Considerations during the COVID-19 Crisis | Iddir M, Brito A, Dingeo G, Fernandez Del Campo SS, Samouda H, La Frano MR, Bohn T. | 2020 |
| Systematic review/meta-analysis/review | Commentary: Myths and facts on vitamin D amidst the COVID-19 pandemic | Chakhtoura M, Napoli N, El Hajj Fuleihan G. | 2020 |
| Systematic review/meta-analysis/review | Vitamin-D and COVID-19: do deficient risk a poorer outcome? | Mitchell F. | 2020 |
| Systematic review/meta-analysis/review | Lungs as target of COVID-19 infection: Protective common molecular mechanisms of vitamin D and melatonin as a new potential synergistic treatment | Martín Giménez VM, Inserra F, Tajer CD, Mariani J, Ferder L, Reiter RJ, Manucha W. | 2020 |
| Systematic review/meta-analysis/review | Mechanism of inflammatory response in associated comorbidities in COVID-19 | de Lucena TMC, da Silva Santos AF, de Lima BR, de Albuquerque Borborema ME, de Azevêdo Silva J. | 2020 |
| Systematic review/meta-analysis/review | Myth Busters: Dietary Supplements and COVID-19 | Adams KK, Baker WL, Sobieraj DM. | 2020 |
| Systematic review/meta-analysis/review | Does Vitamin D play a role in the management of Covid-19 in Brazil? | Glicio, El James, | 2020 |
| Systematic review/meta-analysis/review | Strengthening the Immune System and Reducing Inflammation and Oxidative Stress through Diet and Nutrition: Considerations during the COVID-19 Crisis | Roya Ghasemian1, Amir Shamshirian2,3*, Keyvan Heydari et al. | 2020 |
| Systematic review/meta-analysis/review | Commentary: Myths and facts on vitamin D amidst the COVID-19 pandemic | Weir EK, Thenappan T, Bhargava M, Chen Y. | 2020 |
| Systematic review/meta-analysis/review | Vitamin-D and COVID-19: do deficient risk a poorer outcome? | Ribeiro H, Santana KVS, Oliver SL, Rondó PHC, Mendes MM, Charlton K, Lanham-New S. | 2020 |
| Systematic review/meta-analysis/review | Lungs as target of COVID-19 infection: Protective common molecular mechanisms of vitamin D and melatonin as a new potential synergistic treatment | Chakhtoura M, Napoli N, El Hajj Fuleihan G. | 2020 |
| Systematic review/meta-analysis/review | Mechanism of inflammatory response in associated comorbidities in COVID-19 | Speeckaert MM, Delanghe JR. | 2020 |
| Systematic review/meta-analysis/review | Myth Busters: Dietary Supplements and COVID-19 | Martín Giménez VM, Inserra F, Tajer CD, Mariani J, Ferder L, Reiter RJ, Manucha W. | 2020 |
| Systematic review/meta-analysis/review | Vitamin D Level of Mild and Severe Elderly Cases of COVID-19: A Preliminary Report (May 5, 2020) | Silberstein M. | 2020 |

| **Type** | **Title** | **Authors** | **Year** |
| --- | --- | --- | --- |
| RCT | Effect of monthly high-dose vitamin D supplementation on acute respiratory infections in older adults: A randomized controlled trial. | Camargo CA, Sluyter J, Stewart AW et al. (104) | 2019 |
| RCT | Effect of Vitamin D supplementation to reduce respiratory infections in children and adolescents in Vietnam: A randomized controlled trial. | Loeb M, Dang AD, Thiem VD, et al. (97) | 2019 |
| RCT | Winter cholecalciferol supplementation at 55degreeN has little effect on markers of innate immune defence in healthy children aged 4-8 years: a secondary analysis from a randomized controlled trial. | Hauger H, Ritz C, Mortensen C  et al. (92) | 2019 |
| RCT | Vitamin D and Acute Respiratory Infections—The PODA Trial | Aloia J & Mikhail M(105) | 2019 |
| RCT | Randomized phase 2 trial of monthly vitamin D to prevent respiratory complications in children with sickle cell disease. | Lee MT, Kattan M, Fennoy I,  et al. (98) | 2018 |
| RCT | Therapeutic effect of vitamin D in acute lower respiratory infection: A randomized controlled trial. | Somnath SH, Biswal N, Chandrasekara, Jagadisan B, Bobby (62) | 2017 |
| RCT | High-Dose Monthly Vitamin D for Prevention of Acute Respiratory Infection in Older Long-Term Care Residents: A Randomized Clinical Trial. | Ginde AA, Blatchford P, Breese K, et al.(136) | 2017 |
| RCT | Effect of vitamin D3 on the antimicrobial activity of human airway surface liquid: preliminary results of a randomised placebo-controlled double-blind trial. | Vargas-Buonfiglio LG, Cano M, Pezzulo AA, et al.(76) | 2017 |
| RCT | Vitamin D supplementation during pregnancy and infancy reduces aeroallergen sensitization: a randomized controlled trial. | Grant CC, Crane J, Mitchell EA, et al.(94) | 2016 |
| RCT | The Vitamin D Assessment (ViDA) Study: design of a randomized controlled trial of vitamin D supplementation for the prevention of cardiovascular disease, acute respiratory infection, falls and non-vertebral fractures. | Scragg R, Waayer D, Stewart AW, et al.(137) | 2016 |
| RCT | The effect of 14 weeks of vitamin D3 supplementation on antimicrobial peptides and proteins in athletes. | He C, Fraser W, Tang J, et al.(88) | 2016 |
| RCT | Double-blind randomised controlled trial of vitamin D3 supplementation for the prevention of acute respiratory infection in older adults and their carers (ViDiFlu). | Martineau AR, Hanifa Y, Witt KD et al.(108) | 2015 |
| RCT | Reduced primary care respiratory infection visits following pregnancy and infancy vitamin D supplementation: a randomised controlled trial. | Grant CC, Kaur S, Waymouth Eet al. (100) | 2014 |
| RCT | Vitamin D supplementation and upper respiratory tract infections in adolescent swimmers: a randomized controlled trial. | Dubnov-Raz G, Rinat B, Hemila H  et al. (106) | 2015 |
| RCT | Prenatal vitamin d supplementation and child respiratory health: a randomised controlled trial. | Goldring ST(93) | 2013 |
| RCT | Randomized trial of vitamin D supplementation and risk of acute respiratory infection in Mongolia. | Camargo CA Jr, Ganmaa D  Frazier AL,et al.(101) | 2012 |
| intervention but not RCT | Vitamin D Supplementation in COVID-19 Patients: A Clinical Case Series | Ohaegbulam KC, Swalih M, Patel P, Smith MA, Perrin R. | 2020 |

| **Article type** | **Title** | **Authors** | **Year** |
| --- | --- | --- | --- |
| Obs. Study | A cross sectional analysis of the role of the antimicrobial peptide cathelicidin in lung function impairment within the ALIVE cohort. | Lambert AA, Kirk GD, Astemborski J, et al.(75) | 2014 |
| Obs. Study | Vitamin D Status at the Time of Hospitalization for Bronchiolitis and Its Association with Disease Severity. | Vo P, Koppel C, Espinola JA,  et al. (54) | 2018 |
| Obs. Study | Circulating 25-hydroxyvitamin D, nasopharyngeal microbiota, and bronchiolitis severity. | Toivonen L, Hasegawa K, Ajami N, et al.(138) | 2018 |
| Obs. Study | Effect of vitamin D deficiency in Korean patients with acute respiratory distress syndrome. | Park S, Lee M, Lim C, Koh Y(139) | 2018 |
| Obs. Study | Circulating 25-hydroxyvitamin D, nasopharyngeal airway metabolome, and bronchiolitis severity. | Hasegawa K, Stewart C, Celedon J, Mansbach JM  Tierney C, Camargo CA Jr(140) | 2018 |
| Obs. Study | Serum 25-hydroxyvitamin D levels in patients with Granulomatosis with Polyangiitis: association with respiratory infection | Perez MO, Oliveira RM  Levy-Neto M, Caparbo VF, Pereira RMR(141) | 2017 |
| Obs. Study | Vitamin D Deficiency Is Not Associated With Growth or the Incidence of Common Morbidities Among Tanzanian Infants | Sudfeld CR, Manji KP, Smith ER  et al.(141) | 2017 |
| Obs. Study | 25-Hydroxyvitamin D supplementation and health-service utilization for upper respiratory tract infection in young children. | Omand JA, To T, O'Connor DL, Parkin PC, Birken CS, Thorpe KE, Maguire JL(90) | 2017 |
| Obs. Study | Low Retinol-Binding Protein and Vitamin D Levels Are Associated with Severe Outcomes in Children Hospitalized with Lower Respiratory Tract Infection and Respiratory Syncytial Virus or Human Metapneumovirus Detection. | Hurwitz JL, Jones BG, Penkert RR, et al.(53) | 2017 |
| Obs. Study | Higher serum 25(OH)D concentration is associated with lower risk of chronic otitis media with effusion: a case-control study. | Walker R, Bartley J, Camargo C, Flint D, Thompson J(142) | 2017 |
| Obs. Study | Association Between Serum Vitamin D and All-Cause and Cause-Specific Death in a General Japanese Population - The Hisayama Study. | Umehara K, Mukai N, Hata J  et al. (58) | 2017 |
| Obs. Study | Low cord-serum 25-hydroxyvitamin D levels are associated with poor lung function performance and increased respiratory infection in infancy. | [Lai](https://www.ncbi.nlm.nih.gov/pubmed/?term=Lai%20SH%5BAuthor%5D&cauthor=true&cauthor_uid=28267792),  [Liao](https://www.ncbi.nlm.nih.gov/pubmed/?term=Liao%20SL%5BAuthor%5D&cauthor=true&cauthor_uid=28267792),  [Tsai](https://www.ncbi.nlm.nih.gov/pubmed/?term=Tsai%20MH%5BAuthor%5D&cauthor=true&cauthor_uid=28267792), et al.(143) | 2017 |
| Obs. Study | Cord blood vitamin D and the risk of acute lower respiratory infection in Indigenous infants in the Northern Territory. | Binks M, Smith-Vaughan, Marsh R, Chang A, Andrews R(144) | 2016 |
| Obs. Study | Association of vitamin D deficiency with acute lower respiratory tract infections in newborns. | Dinlen, Zenciroglu, Beken, Dursun, Dilli & Okumus(145) | 2015 |
| Obs. Study | Correlation of serum vitamin A, D, and E with recurrent respiratory infection in children. | Zhang J, Sun RR, Yan ZX, Yi WX, Yue B (45) | 2019 |
| Obs. Study | Association between serum vitamin D, retinol and zinc status, and acute respiratory infections in underweight and normal-weight children aged 6-24 months living in an urban slum in Bangladesh. | Ahmed A, Ahmed T Soares-Magalhaes R, et al.(146) | 2016 |
| Obs. Study | Association between prehospital vitamin D status and incident acute respiratory failure in critically ill patients: a retrospective cohort study. | Thicket D, Moromizato T, Litonjua A, et al.(56) | 2015 |
| Obs. Study | Vitamin D deficiency and risk of acute lung injury in severe sepsis and severe trauma: a case-control study. | Barnett N, Zhao Z  Koyama T, et al. (48) | 2014 |
| Obs. Study | Vitamin D intake in young children with acute lower respiratory infection | Leis KS, McNally JD, Montgomery MR, Sankaran K, Karunanayake C, Rosenberg A(147) | 2012 |
| Obs. Study | Vitamin D status and acute respiratory infection: cross sectional results from the United States National Health and Nutrition Examination Survey, 2001-2006. | Monlezun DJ, Bittner EA, Christopher KB, Camargo CA  Quraishi SA(43) | 2015 |
| Obs. Study | Vitamin D status and hospitalisation for childhood acute lower respiratory tract infections in Nigeria. | Ahmed P, Babaniyi I,  Yusuf KK, et al.(148) | 2015 |
| Obs. Study | Vitamin D insufficiency among hospitalised children in the Northern Territory. | Binks Smith-Vaughan HC, Bar-Zeev N, Chang AB, Andrews RM(149) | 2014 |
| Obs. Study | Influence of vitamin D status on respiratory infection incidence and immune function during 4 months of winter training in endurance sport athletes. | He CS, Handzlik M, Fraser WD, et al(57) | 2013 |
| Obs. Study | The role of vitamin D supplementation in the risk of developing pneumonia: three independent case-control studies. | Remmelts H, Spoorenberg S,  Oosterheert J, Bos W, de Groot MC, van de Garde (150) | 2013 |
| Obs. Study | Association of Vitamin D Status and Other Clinical Characteristics With COVID-19 Test Results | Meltzer DO, Best TJ, Zhang H, Vokes T, Arora V, Solway J. | 2020 |
| Obs. Study | Vitamin D Insufficiency and Deficiency and Mortality from Respiratory Diseases in a Cohort of Older Adults: Potential for Limiting the Death Toll during and beyond the COVID-19 Pandemic? | Brenner H, Holleczek B, Schöttker B. | 2020 |
| Obs. Study | Revisiting the role of vitamin D levels in the prevention of COVID-19 infection and mortality in European countries post infections peak | Singh S, Kaur R, Singh RK. | 2020 |
| Obs. Study | A Look Behind the Scenes at COVID-19: National Strategies of Infection Control and Their Impact on Mortality | Haj Bloukh S, Edis Z, Shaikh AA, Pathan HM. | 2020 |
| Obs. Study | COVID-19 fatalities, latitude, sunlight, and vitamin D | Whittemore PB. | 2020 |
| Obs. Study | Serum calcium as a biomarker of clinical severity and prognosis in patients with coronavirus disease 2019 | Sun JK, Zhang WH, Zou L, Liu Y, Li JJ, Kan XH, Dai L, Shi QK, Yuan ST, Yu WK, Xu HY, Gu W, Qi JW. | 2020 |
| Obs. Study | Greater risk of severe COVID-19 in Black, Asian and Minority Ethnic populations is not explained by cardiometabolic, socioeconomic or behavioural factors, or by 25(OH)-vitamin D status: study of 1326 cases from the UK Biobank | Raisi-Estabragh Z, McCracken C, Bethell MS, Cooper J, Cooper C, Caulfield MJ, Munroe PB, Harvey NC, Petersen SE. | 2020 |
| Obs. Study | Vitamin D concentrations and COVID-19 infection in UK Biobank | Hastie CE, Mackay DF, Ho F, Celis-Morales CA, Katikireddi SV, Niedzwiedz CL, Jani BD, Welsh P, Mair FS, Gray SR, O'Donnell CA, Gill JM, Sattar N, Pell JP. | 2020 |
| Obs. Study | Perspective: improving vitamin D status in the management of COVID-19 | Ebadi M, Montano-Loza AJ. | 2020 |
| Obs. Study | 25-Hydroxyvitamin D Concentrations Are Lower in Patients with Positive PCR for SARS-CoV-2 | D'Avolio A, Avataneo V, Manca A, Cusato J, De Nicolò A, Lucchini R, Keller F, Cantù M. | 2020 |
| Obs. Study | The role of vitamin D in the prevention of coronavirus disease 2019 infection and mortality | Ilie PC, Stefanescu S, Smith L. | 2020 |
| Obs. study | Vitamin D Deficiency and ARDS after SARS-CoV-2 Infection | Faul JL, Kerley CP, Love B, O'Neill E, Cody C, Tormey W, Hutchinson K, Cormican LJ, Burke CM. | 2020 |
| Obs. Study | Relationship of maternal vitamin D level with maternal and infant respiratory disease. | CarrollKN, Gebretsadik T, Larkin EK, et al. (51) | 2011 |
| Obs. Study  Children | Serum vitamin D concentrations and associated severity of acute lower respiratory tract infections in Japanese hospitalized children. | Inamo Y, Hasegawa M, Saito K, et al.(52) | 2011 |
| Obs. Study  Adults | Active vitamin D and acute respiratory infections in dialysis patients. | Tsujimoto Y, Tahara H,, Shoji T, et al.(151) | 2011 |
| Obs. Study  Infant | Cord-blood 25-hydroxyvitamin D levels and risk of respiratory infection, wheezing, and asthma. | Camargo CA Jr, Ingham T, Wickens K, et al.(152) | 2011 |
| Obs. Study  Children | Vitamin D status and acute lower respiratory infection in early childhood in Sylhet, Bangladesh. | Roth, D E, Shah, R, Black, R E, Baqui, A H(153) | 2010 |
| Obs. Study  Children | Vitamin D deficiency in young children with severe acute lower respiratory infection. | McNally J, Leis K, Matheson L, Karuananyake, Sankaran K, Rosenberg A(50) | 2009 |
| Obs. Study  Infants | Association of subclinical vitamin D deficiency in newborns with acute lower respiratory infection and their mothers. | Karatekin G, Kaya A, Salihoglu O, Balci H, Nuhoglu A(59) | 2009 |
| Obs. Study  Adults | An association of serum vitamin D concentrations < 40nmol/L with acute respiratory tract infection in young Finnish men. | Laaksi I, Ruohola JP, Tuohimaa P, et al.(154) | 2007 |
| Obs. Study  Children | Association of subclinical vitamin D deficiency with severe acute lower respiratory infection in Indian children under 5 y. | Wayse V, Yousafzai A, Mogale K & Filteau S(49) | 2004 |

| **Type** | **Title** | **Authors** | **Year** |
| --- | --- | --- | --- |
| Experimental | Vitamin D attenuates lung injury via stimulating epithelial repair, reducing epithelial cell apoptosis and inhibits TGF-beta induced epithelial to mesenchymal transition | Zheng S, Yang J,Hu X et al.(64) | 2020 |
| Experimental | Vitamin D receptor genotype influences risk of upper respiratory infection. | Jolliffe DA, Greiller CL, Mein CA, et al.(66) | 2018 |
| Experimental | Vitamin D alleviates lipopolysaccharide-induced acute lung injury via regulation of the renin-angiotensin system. | Xu J, Yang J, Chen J, Luo Q, Zhang Q, Zhang H(65) | 2017 |
| Experimental | Vitamin D/VDR signalling attenuates lipopolysaccharide-induced acute lung injury by maintaining the integrity of the pulmonary epithelial barrier. | Yong-Yan Shi, Tian-Jing Liu, Jian‑Hua Fu, et al.(155) | 2015 |
| Experimental | Vitamin D deficiency contributes directly to the acute respiratory distress syndrome (ARDS). | Dancer RC, Parekh D, Lax S, et al.(3) | 2015 |

| **Type** | **Title** | **Authors** | **Year** |
| --- | --- | --- | --- |
| Editorial | Editorial: low population mortality from COVID-19 in countries south of latitude 35 degrees North - supports vitamin D as a factor determining severity. | Rhodes JM, Subramanian S, Laird E, Anne Kenny R(6) | 2020 |
| Special report | Early nutritional supplementation in non-critically ill patients hospitalized for the 2019 novel coronavirus disease (COVID-19): Rationale and feasibility of a shared pragmatic protocol. | Caccialanza R, Laviano A, Lobascio F, et al.(130) | 2020 |
| Letter | Considerations for obesity, vitamin D, and physical activity amidst the COVID-19 pandemic. | Carter SJ, Baranauskas MN  Fly AD(70) | 2020 |
| Letter | Letter: Covid-19, and vitamin D. | Panarese A, Shahini E | 2020 |
| Letter | Letter: Covid-19 and vitamin D-authors' reply | Tian Y, Rong L(71) | 2020 |
| Letter | Vitamin D and coronavirus: a new field of use [ltalian] | Maestri E, Formoso G, Da Cas R, Mammarella F, Guerrizio MA,  Trotta F(156) | 2020 |
| Position statement | Current vitamin D status in European and Middle East countries and strategies to prevent vitamin D deficiency: a position statement of the European Calcified Tissue Society. | Lips P, Cashman KD, Lamberg-Allardt C, et al.(8) | 2019 |
| Letter | COVID-19 and Vitamin D- Is there a link and an opportunity for intervention? | Jakovac H(62) | 2020 |
| Letter | Vitamin D, Covid-19 and Children. | Molloy E & Murphy N(77) | 2020 |
| Comment | Optimisation of Vitamin D Status for Enhanced Immuno-protection Against Covid-19. | McCartney DM, Byrne DG(4) | 2020 |
| Comment | Vitamin D for Acute Respiratory Distress Syndrome: Sunshine or Shade? | Ahmad S(157) | 2018 |
| Letter | Reply to: Analysis Leads to Unreliable Results in Study of Vitamin D and Acute Respiratory Infection | Ginde AA, Blatchford P(158) | 2017 |
| Letter | Analysis Leads to Unreliable Results in Study of Vitamin D and Acute Respiratory Infection. | Beale DJ(159) | 2017 |
| Comment | Vitamin D supplementation effective in preventing acute respiratory tract infections. | Anonymous | 2017 |
| Editorial | Vitamin D dosing for infectious and immune disorders. | Weiss ST, Litonjua AA(160) | 2015 |
| Editorial | 'Curiouser and curiouser': the role of vitamin D in the prevention of acute respiratory infection. | Martineau AR(161) | 2015 |
| Editorial | Pandemic preparedness for swine flu influenza in the United States. | Edlich R, Mason S, Dahlstrom J, Swainston E, Long W, Gubler K(162) | 2009 |
| Editorial/letter/comment | Vitamin D supplementation to prevent COVID-19 in patients with COPD: a research perspective | Chaabouni M, Feki W, Chaabouni K, Kammoun S. | 2020 |
| Editorial/letter/comment | Reply to Jakovac and to Rocha et al.: Can vitamin D prevent or manage COVID-19 illness? | Slominski AT, Slominski RM, Goepfert PA, Kim TK, Holick MF, Jetten AM, Raman C. | 2020 |
| Editorial/letter/comment | Vitamin D for COVID-19: a case to answer? | Martineau AR, Forouhi NG. | 2020 |
| Editorial/letter/comment | Vitamin D Supplementation During the COVID-19 Pandemic | Siuka D, Pfeifer M, Pinter B. | 2020 |
| Editorial/letter/comment | Reply to Jakovac: COVID-19, vitamin D, and type I interferon | Gauzzi MC, Fantuzzi L. | 2020 |
| Editorial/letter/comment | Comments on: The role of vitamin D in the prevention of coronavirus disease 2019 infection and mortality | Maruotti A, Belloc F, Nicita A. | 2020 |
| Editorial/letter/comment | COVID-19 and vitamin D deficiency, a fatal combination? | van der Meulen J. | 2020 |
| Editorial/letter/comment | The D-side of COVID-19: musculoskeletal benefits of vitamin D and beyond | Tramontana F, Napoli N, El-Hajj Fuleihan G, Strollo R. | 2020 |
|  |  |  |  |
| Editorial/letter/comment | Covid-19, Cocooning and Vitamin D Intake Requirements | McKenna MJ, Flynn MAT. | 2020 |
| Editorial/letter/comment | Photobiomodulation: Shining Light on COVID-19 | Fernandes AB, de Lima CJ, Villaverde AGJB, Pereira PC, Carvalho HC, Zângaro RA. | 2020 |
| Editorial/letter/comment | COVID-19 - Does This Disease Kill Due to Imbalance of the Renin Angiotensin System (RAS) Caused by Genetic and Gender Differences in the Response to Viral ACE 2 Attack? | Arnold RH. | 2020 |
| Editorial/letter/comment | The possible pathophysiology mechanism of cytokine storm in elderly adults with COVID-19 infection: the contribution of "inflame-aging" | Meftahi GH, Jangravi Z, Sahraei H, Bahari Z. | 2020 |
| Editorial/letter/comment | Diet Supplementation, Probiotics, and Nutraceuticals in SARS-CoV-2 Infection: A Scoping Review | Infusino F, Marazzato M, Mancone M, Fedele F, Mastroianni CM, Severino P, Ceccarelli G, Santinelli L, Cavarretta E, Marullo AGM, Miraldi F, Carnevale R, Nocella C, Biondi-Zoccai G, Pagnini C, Schiavon S, Pugliese F, Frati G, d'Ettorre G. | 2020 |
| Editorial/letter/comment | Sub-continental Atmosphere and Inherent Immune System may have Impact on Novel Corona Virus' 2019 (nCovid-19) Prevalence in South East Asia | Khalil I, Barma P. | 2020 |
| Editorial/letter/comment | Does vitamin D deficiency increase the severity of COVID-19? | Weir EK, Thenappan T, Bhargava M, Chen Y. | 2020 |
| Editorial/letter/comment | Vitamin D Supplementation in Influenza and COVID-19 Infections Comment on: "Evidence that Vitamin D Supplementation Could Reduce Risk of Influenza and COVID-19 Infections and Deaths" Nutrients 2020, 12(4), 988 | Kow CS, Hadi MA, Hasan SS. | 2020 |
| Editorial/letter/comment | Evidence That Vitamin D Supplementation Could Reduce Risk of Influenza and COVID-19 Infections and Deaths Nutrients 2020, 12(4), | Grant WB, Baggerly CA, Lahore H. | 2020 |
| Editorial/letter/comment | Association between low vitamin D and COVID-19: don't forget the vitamin D binding protein | Speeckaert MM, Delanghe JR. | 2020 |
| Editorial/letter/comment | Vitamin D can prevent COVID-19 infection-induced multiple organ damage | Aygun H. | 2020 |
| Editorial/letter/comment | Reply to Jakovac: About COVID-19 and vitamin D | Facchiano A, Facchiano A, Bartoli M, Ricci A, Facchiano F. | 2020 |
| Editorial/letter/comment | 'Scientific Strabismus' or two related pandemics: coronavirus disease and vitamin D deficiency | Kara M, Ekiz T, Ricci V, Kara Ö, Chang KV, Özçakar L. | 2020 |
| Editorial/Letter/Comment | Vitamin D: A simpler alternative to tocilizumab for trial in COVID-19? | Silberstein M. | 2020 |
| Editorial/letter/comment | Editorial: low population mortality from COVID-19 in countries south of latitude 35 degrees North-supports vitamin D as a factor determining severity. Authors' reply | Garg M, Al-Ani A, Mitchell H, Hendy P, Christensen B. | 2020 |
| Editorial/letter/comment | Vitamin D Supplementation in Influenza and COVID-19 Infections Comment on: "Evidence that Vitamin D Supplementation Could Reduce Risk of Influenza and COVID-19 Infections and Deaths" Nutrients 2020, 12(4), 988 | Davies G, Garami AR, Byers J. | 2020 |
| Editorial/letter/comment | Reply: "Vitamin D Supplementation in Influenza and COVID-19 Infections. Comment on: Evidence That Vitamin D Supplementation Could Reduce Risk of Influenza and COVID-19 Infections and Deaths Nutrients 2020, 12(4), 988" | De Smet D, De Smet K, Herroelen P, Gryspeerdt S, Martens GA. | 2020 |
| Editorial/letter/comment | Association between low vitamin D and COVID-19: don't forget the vitamin D binding protein | Kow CS, Hadi MA, Hasan SS. | 2020 |
| Editorial/letter/comment | Vitamin D can prevent COVID-19 infection-induced multiple organ damage | Grant WB, Baggerly CA, Lahore H. | 2020 |
| Editorial/letter/comment | Reply to Jakovac: About COVID-19 and vitamin D | Iddir M, Brito A, Dingeo G, Fernandez Del Campo SS, Samouda H, La Frano MR, Bohn T. | 2020 |
| Editorial/letter/comment | 'Scientific Strabismus' or two related pandemics: coronavirus disease and vitamin D deficiency | de Lucena TMC, da Silva Santos AF, de Lima BR, de Albuquerque Borborema ME, de Azevêdo Silva J. | 2020 |
| Editorial/Letter/Comment | Vitamin D: A simpler alternative to tocilizumab for trial in COVID-19? | Ebadi M, Montano-Loza AJ. | 2020 |
| Editorial/letter/comment | Editorial: low population mortality from COVID-19 in countries south of latitude 35 degrees North-supports vitamin D as a factor determining severity. Authors' reply | D'Avolio A, Avataneo V, Manca A, Cusato J, De Nicolò A, Lucchini R, Keller F, Cantù M. | 2020 |
| Editorial/letter/comment | Vitamin D Supplementation Could Possibly Improve Clinical Outcomes of Patients Infected with Coronavirus-2019 (COVID-2019); April 9, 2020. | Adams KK, Baker WL, Sobieraj DM. | 2020 |
| Editorial/letter/comment | Evidence Supports a Causal Model for Vitamin D in COVID-19 Outcomes. | Kara M, Ekiz T, Ricci V, Kara Ö, Chang KV, Özçakar L. | 2020 |
| Editorial/letter/comment | Vitamin D deficiency as risk factor for severe COVID-19: a convergence of two pandemics, | Ilie PC, Stefanescu S, Smith L. | 2020 |
